# Trade-offs between mobility restrictions and transmission of SARS-CoV-2

**DOI:** 10.1098/rsif.2020.0936

**Published:** 2021-02-24

**Authors:** Martijn Gösgens, Teun Hendriks, Marko Boon, Wim Steenbakkers, Hans Heesterbeek, Remco van der Hofstad, Nelly Litvak

**Affiliations:** ^1^ Department of Mathematics and Computer Science, Eindhoven, University of Technology, Eindhoven, Netherlands; ^2^ Mezuro, Weesp, Netherlands; ^3^ Population Health Sciences, University of Utrecht, Utrecht, Netherlands; ^4^ Faculty of Electrical Engineering, Mathematics and Computer Science, University of Twente, Enschede, Netherlands

**Keywords:** epidemiology, compartmental models, SARS-CoV-2, mobility restrictions, simulation study

## Abstract

In their response to the COVID-19 outbreak, governments face the dilemma to balance public health and economy. Mobility plays a central role in this dilemma because the movement of people enables both economic activity and virus spread. We use mobility data in the form of counts of travellers between regions, to extend the often-used SEIR models to include mobility between regions. We quantify the trade-off between mobility and infection spread in terms of a single parameter, to be chosen by policy makers, and propose strategies for restricting mobility so that the restrictions are minimal while the infection spread is effectively limited. We consider restrictions where the country is divided into regions, and study scenarios where mobility is allowed within these regions, and disallowed between them. We propose heuristic methods to approximate optimal choices for these regions. We evaluate the obtained restrictions based on our trade-off. The results show that our methods are especially effective when the infections are highly concentrated, e.g. around a few municipalities, as resulting from superspreading events that play an important role in the spread of COVID-19. We demonstrate our method in the example of the Netherlands. The results apply more broadly when mobility data are available.

## 1. Introduction

The pandemic of COVID-19, caused by the coronavirus SARS-CoV-2, had, by mid-November 2020, infected more than 50 million people in over 200 countries and led to more than a million deaths. It is unlikely that the spread can be fully controlled in the near future and without the deployment of effective vaccines. Strategies are aimed at curbing exponential growth in case numbers and hospitalizations, predominantly to keep national health systems from becoming overburdened and to reduce infection pressure for people with a high risk of severe outcomes. Such strategies are limited to personal protection and hygiene, social distancing measures, reducing contacts and mixing/mobility. Although the virus is present globally, all countries implement their own strategies and sets of measures.

At any given moment in the outbreak, there is a mix of countries and regions where the virus is temporarily under control, countries where the epidemic is decreasing and countries where the epidemic is increasing. After an initial peak in cases, countries remain at risk for second and subsequent peaks, even when no cases are reported in the country for long periods of time. As in principle everybody is susceptible to some degree, not reaching herd immunity after the initial wave of infection leaves a large susceptible population that can sustain subsequent outbreaks [1]. These new outbreaks can be triggered by infected individuals entering the country from outside, as a result of increased global mobility. Nationally, sustained transmission at relatively low levels can lead to new large (exponentially growing) outbreaks after the initial peak because control measures are relaxed or behaviour changes with respect to (social, temporal and spatial) mixing and personal protection/social distancing. Mixing increases the number of new contact opportunities that an infected individual has in the population and reduced effectiveness of personal protection and social distancing increases the probability per contact of transmission. Combined, these effects can lead to more transmission. Increased mixing not only reflects larger groups of individuals but also reflects contacts with individuals from a larger geographical range, allowing infected individuals to have contacts with people from regions where infection pressure may hitherto be (very) low, causing clusters of cases in new areas.

Mobility between areas plays a potentially important role in increasing transmission, but measures aimed at restricting mobility also have a potentially large social and economic impact: mobility and economic activity are often studied as two sides of the same coin [2]. Where, in the initial wave of infection, countries to a large extent imposed national mobility restrictions, the containment strategies for preventing subsequent waves of infection can perhaps be achieved by more regional or local mobility restrictions. This has the advantage of reducing the social and economic burden on society, but also has the risk that the restrictions may not be sufficiently effective and need to be scaled-up after all to a national level at some later point in time. It is, however, unclear how one could gauge the effectiveness of regional restrictions based on realistic mobility patterns specific to the country, balancing trade-offs between mobility and transmission. It is also unclear how large a ‘region’ should be for effective containment and how different choices for recognizable regions (for example, administrative regions such as provinces, large cities, or postal code regions).

In this paper, we provide a framework to evaluate the effectiveness of regional strategies aimed at restricting mobility, allowing for a range of choices of how regions are characterized, using the Netherlands as a case study. This is essential to be able to determine the scale at which interventions can be effectively imposed or lifted and addresses one of a range of key modelling questions for COVID-19 and future pandemic outbreaks [3]. We base the framework on actual mobility patterns in the Netherlands. We distinguish between extreme situations where infection is distributed evenly between areas and situations where infection is highly concentrated in a restricted area, for example as a result of a superspreading event. We show that regions defined on the basis of mobility patterns provide better strategies than regions based on administrative characteristics, and that focusing on administrative regions therefore leads to sub-optimal strategies. We also quantify and explore the nonlinear relation between mobility and outbreak size for a range of choices of trade-off between mobility and transmission.

## 2. Methodology

In this section, we describe the overview of our approach. Given a certain set of regions, we envision a situation where mobility is allowed within the region, but mobility between the regions is not allowed. The main aim of this paper is to devise regions that allow for as much mobility as possible, yet restrict infections as much as possible. For this, we need to strike a careful balance between mobility and infections, which we formalize in terms of a trade-off parameter that policymakers need to impose.

This section is organized as follows. We start in §2.1 by specifying the kind of mobility strategies that we consider in this work. Next, in §2.2, we introduce a way to quantify the performance of such strategies, by formulating the trade-off between mobility and infection spread as an explicit optimization problem over the various choices of regions described in §2.1. This trade-off is described in terms of the number of infections, for which we rely on an SEIR model that we introduce in §2.3, and mobility. In our SEIR model, the infections are described in terms of compartments that correspond to particular regions, and the infection is spread between regions by mobility between them. Our SEIR model takes such mobility into account, and relies on mobility information originating from telecommunication data.

The optimization problem that formalizes our trade-off between mobility and infection containment is inspired by community-detection algorithms, and is complex to solve explicitly. In §2.4, we describe how to rigorously and heuristically analyse such problems. In particular, we provide heuristics that generate strategies with high performance. We close this section by describing how the various divisions in regions can be evaluated in §2.5.

### 2.1. Strategies for mobility restrictions

We consider mobility restriction strategies of the following kind: given a division of the country into regions, we consider the scenario where movement is allowed *within* these regions, and disallowed *between* these regions. We represent the country by a set of *atomic areas* A. These areas are considered the smallest possible geographical units between which it is feasible to enforce mobility restrictions. Then, a region is represented by a subset D⊆A between which mobility is allowed. Finally, a division is represented by a partition $D=\{D_{1},\ldots ,D_{\left|D\right|}\}$. In our use case, we consider Dutch municipalities to be atomic areas.

Many administrative divisions that might serve as examples for regions D already exist. For example, we could use the divisions of the Netherlands and its municipalities into its 12 provinces, or 25 so-called security regions. An advantage of using such divisions is that they are already known so that it may be easier to communicate, and thus enforce, mobility restrictions based on them to the broad public. However, a disadvantage is that these divisions have been historically determined by decisions of governance, and thus their borders do not necessarily effectively reflect the actual movement of people throughout the country. As a result, mobility restrictions based on them may not be the most effective.

An illustrative example is the province of Flevoland (equal to the security region Flevoland). Almere, the most populous city of Flevoland, lies close to Amsterdam, where many of Almere's citizens work. Our mobility data show that more than 90% of the mobility leaving Almere also leaves the province of Flevoland, as can be seen in figure 4. Therefore, choosing the division into provinces or security regions would disproportionately affect the people of Almere in terms of mobility. In §2.4, we provide a method to obtain divisions based on the mobility data, by applying community detection methods. In figure 4, we see that divisions obtained by this method do consistently place Almere in the same region as Amsterdam.

Another disadvantage of using existing administrative divisions is that these are, by their very definition, inflexible and hence cannot be tailored to the specific epidemiological situation. In §2.4, we provide a method to obtain divisions that do take epidemiological information into account.

### 2.2. Objective

On the one hand, freedom of movement has both economic and intrinsic value. On the other hand, it also facilitates the spread of the disease. In essence, the problem for control is to find a trade-off between mobility and infection containment, given the epidemiological characteristics and normal mobility patterns. In this section, we provide a way to *formalize* this trade-off to allow its characterization.

#### 2.2.1. Trade-off parameter

To formalize the trade-off between public health and mobility, we introduce a trade-off parameter *γ*. This parameter can be interpreted as the number of movements between areas that we are willing to restrict in order to prevent the occurrence of a *single* further infection. A higher value for *γ* thus favours more severe restrictions. The choice for this trade-off parameter reflects societal values and is hence a political choice that should be made by politicians or policy makers. Therefore, we refrain from giving advice about a specific suitable value, but instead provide a method that advises a strategy given a choice for *γ*.

#### 2.2.2. Time horizon

Suppose that a certain set of mobility restrictions is in place. We consider some time horizon *H* representing the number of days that the restriction will be in place, and count the number of infections and movements before this horizon. This time horizon should thus not be too long, as it coincides with the duration that restrictions are in place and sometimes one needs to quickly respond to changes in the infection spread. Due to the delay to go from a contact moment to an infection, the time horizon should also not be too short, as otherwise the effect of the imposed restrictions cannot reasonably be observed. In this work, we use a time horizon of 30 days as an example.

At the end of this time horizon, a new division into regions may be chosen based on the status of the epidemic at that moment in time, thus allowing for a dynamical update of the strategy of mobility restrictions.

#### 2.2.3. Objective function

The above considerations lead to the objective function

2.1 $$Q_{\gamma ,H}(D)=M(D;H)-\gamma G(D;H),$$

where M(D;H) and G(D;H) represent the number of movements and infections, respectively, that occur before the time horizon *H*, given a division D. Note that in this formulation the current status of the epidemic, i.e. the number of infectious and susceptible people at the start of the period *H*, is included because the value of G(D;H) depends on that initial status. The objective function (2.1) should be interpreted as follows: given divisions D and $D^{\prime }$, if $Q_{\gamma ,H}(D)>Q_{\gamma ,H}(D^{\prime })$, then D is preferred over $D^{\prime }$ with respect to the trade-off given by *γ*. Hence, the objective function establishes an ordering among the divisions. The goal of this study is to provide a methodology that, given *γ* and *H*, finds a division D with a (locally) optimal $Q_{\gamma ,H}(D)$.

#### 2.2.4. Estimating mobility

The company Mezuro has a platform that produces mobility patterns based on telecom data. It provides information about the average number of people that move between the different municipalities in a given time period; in our case study this is the period from 1 March 2019 up to and including 14 March 2019. Given a division, the mobility between two municipalities in the same region is estimated by the average mobility observed over these two weeks. The mobility between two municipalities in different regions is assumed to be zero, corresponding to full compliance to the restrictions. By using a daily average, we lose the difference between weekdays and weekends. Also, the period on which the average in our mobility data is based is obviously before the COVID-19 outbreak. We interpret this data as being the mobility benchmark: this is what people *would* travel if COVID-19 were not present. Thus, any mobility that is less arises through the governmental and societal measures that are put upon our society.

#### 2.2.5. Estimating infections

Infection dynamics are described by a modified version of a commonly used compartmental SEIR model [4], where people can be either susceptible (S), exposed (E), infectious (I) or removed (R). Exposed people are infected but become infectious after a latent period. Removed people are recovered from the disease, assuming life-long immunity, or passed away caused by the disease. Differential equations govern the rates of infection spread between and within the different compartments. In our model, we capture the spatial component of infection spread, population distribution, and connectivity between different regions. This model is explained in the next section.

### 2.3. Region-based epidemiological model

**Figure 1.**
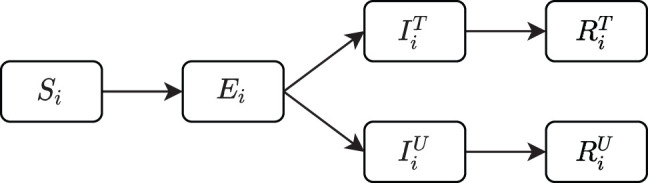
The SEI_2_R_2_ compartmental model within municipality *i*. *U*, *T* stand for ‘Untested’ and ‘Tested’. The rate of new infections *S*_*i*_ → *E*_*i*_ consists of a local part within municipality *i* and a non-local part which depends on infectious people from other municipalities via mobility to and from these municipalities.

We propose a modified SEIR model that incorporates mobility. Firstly, to take into account the spatial component and population distribution, we divide the total population *N* into *n* smaller groups, designating spatial distinct areas with population size *N*_*i*_, i∈[1,2,…,n]=A, such that $N=\sum_{i=1}^{n}N_{i}$. Such an approach has been commonly used in epidemiological models since pioneering work in the mid-nineteen nineties [5–10]. In the context of COVID-19, metapopulation and network models have been used to study effects of mobility restrictions for subdivisions in municipalities and provinces [11–14]. We call the groups *atomic areas*. Using municipalities in the Netherlands would set *n* = 380 (based on 2018). To potentially model control and behavioural effects on mobility better for SARS-CoV-2, we distinguish between tested and untested individuals in infectious and removed compartments, indicated by the superscripts *T* and *U*, respectively. Figure 1 shows the different compartments. Because there are two infectious and two removed compartments, we abbreviate it to SEI_2_R_2_. Others use a similar approach to modelling SARS-CoV-2 [15,16].

To model movements between different atomic areas, we introduce the mobility parameter *M*_*ij*_(*t*), which is interpreted as the number of individuals from atomic area *j* who visit atomic area *i* at time *t*. If areas are taken as municipalities, then we can use Mezuro mobility data as a proxy for *M*_*ij*_. People are assumed to always return home at the end of every day, i.e. visits to other locations are assumed to be brief.

In the SEIR model, an important parameter is the transmission rate *β*. We assume the number of contacts per person per unit time to be independent of the population size, which is called standard incidence. In this case, *β* is the product of the contact rate *c* (contacts per person per unit time) and the transmission probability of the virus ɛ. Our model aims to capture the reduction in contacts a person has when their mobility is restricted. A lower contact rate translates into a lower transmission rate of the virus.

To achieve this in our mobility setting, we split *c* in local contacts within one's atomic area *j*, and non-local contacts resulting from travel to any other another atomic area *i*, as described by the *M*_*ij*_. We assume local contacts to be a fraction *p* of the overall average contact rate; *c*_loc_ = *pc*. In the population of size *N*, the total number of meetings is *cN*/2, assuming a contact is only between two people, of which a fraction *p* is now accounted for locally. The remainder of contacts are made through travelling people who visit other areas and mix with the individuals present there. There are $\sum_{i,j\in A}M_{ij}$ travelling people. We calculate the contacts per travelling person per unit time using

$$c_{\mathrm{mob}}=(1-p)c\frac{N}{2\sum_{i,j\in A}M_{ij}}.$$

In a system where we impose no restriction on mobility, we have an overall average contact rate equal to *c*, but when mobility between regions is restricted, the overall average contact rate decreases.

We define *β*_loc_ = *ɛc*_loc_ and *β*_mob_ = *ɛc*_mob_. We next explain how *β*_loc_ and *β*_mob_ can be incorporated in the SEI_2_R_2_ model. New infections arise by three different mechanisms. (i) Locally, infectious people infect susceptibles within their atomic area *i*, (ii) susceptibles from area *i* visit area *j* and get infected, or (iii) infectious people from area *j* visit area *i* and infect susceptible inhabitants of region *i*. We assume that tested infectious people minimize their contacts to only local contacts, so that they do not play a role in spreading the virus to other areas.

This results in the following mean-field differential equations for the dynamics of the model:

$$\begin{array}{rl} \frac{dS_{i}(t)}{dt}= & -\beta_{\mathrm{loc}}\frac{S_{i}(t)}{N_{i}}(I_{i}^{T}(t)+I_{i}^{U}(t))\\ & -\beta_{\mathrm{mob}}\sum_{\;j\in D}\left(S_{i}(t)\frac{M_{\;ji}}{N_{i}}\frac{I_{\;j}^{U}(t)}{N_{\;j}}+I_{\;j}^{U}(t)\frac{M_{ij}}{N_{\;j}}\frac{S_{i}(t)}{N_{i}}\right),\\ \frac{dE_{i}(t)}{dt}= & \;\beta_{\mathrm{loc}}\frac{S_{i}(t)}{N_{i}}(I_{i}^{T}(t)+I_{i}^{U}(t))\\ & +\beta_{\mathrm{mob}}\frac{S_{i}(t)}{N_{i}}\sum_{\;j\in D}\frac{I_{\;j}^{U}(t)}{N_{\;j}}(M_{\;ji}+M_{ij})-\frac{E_{i}(t)}{\nu },\\ \frac{dI_{i}^{T}(t)}{dt}= & \;a\frac{E_{i}(t)}{\nu }-\frac{I_{i}^{T}(t)}{\omega },\\ \frac{dI_{i}^{U}(t)}{dt}= & \;(1-a)\frac{E_{i}(t)}{\nu }-\frac{I_{i}^{U}(t)}{\omega },\\ \frac{dR_{i}^{T}(t)}{dt}= & \;\frac{I_{i}^{T}(t)}{\omega }\;\\ \mathrm{and}\;\frac{dR_{i}^{U}(t)}{dt}= & \;\frac{I_{i}^{U}(t)}{\omega }. \end{array}$$

Here atomic area *i* is by convention part of region *D*. We have the following parameters: *a* is the fraction of people which gets tested, *ν* is the latent period and *ω* is the duration of the infectious period.

We assume these parameters to be constant. For the fraction of people who get tested this is not entirely realistic: this fraction depends on the test capacity and government regulations, which are subject to change over time. However, for the relatively short time-periods that are simulated, this fraction can reasonably be considered constant. Estimates of all parameters can be found in table 1. Summation over *j* ∈ *D* means we only allow travel from atomic area *i* to and from atomic area *j* if it is within the same partition *D* as atomic area *i*, where D∈D.

**Table 1.** Values of the parameters in our epidemic model.

name		value	source
fraction tested	*a*	1/15	estimated
fraction local contacts	*p*	1/2	estimated
infectious period	*ω*	5 days	Deng *et al.* [17]
latent period	*ν*	4 days	shorter than incubation period [18,19]
basic reproduction number	$R_{0}$	2.5	Li *et al.* [20]
prevention measure efficacy		50%	estimated
effective reproduction number (with prevention measures)	$R_{\mathrm{eff}}$	1.25	50% of $R_{0}$
transmission probability per contact with infectious person	*ɛ*	0.0238	NGM and $R_{\mathrm{eff}}=1.25$
average contact rate (unique persons)	*c*	13.85	Mossung *et al.* [21]
transmission rate via local contacts	*β*_loc_	0.165	*β*_loc_ = *c*_loc_*ɛ*
transmission rate via mobility related contacts	*β*_mob_	0.141	*β*_mob_ = *c*_mob_*ɛ*

It is estimated that the basic reproduction number $R_{0}$ of COVID-19 is in the range [2, 3] in a population without additional measures such as social distancing, sneezing in one's elbow and regular hand-washing [20]. In our model, these measures are not modelled explicitly, but they are accounted for by using an effective reproduction number *R*_eff_ without mobility restrictions smaller than 2. This corresponds to a situation where all mobility is allowed combined with preventive measures being in place, resulting in a lower $R_{\mathrm{eff}}$ and hence a lower *ɛ*. For exposition, we use a value $R_{\mathrm{eff}}=1.25$. For such a value, the effective $R_{\mathrm{eff}}(D)$ including the mobility measures will be smaller than 1 when no mobility is allowed, so that an effective choice of mobility restrictions can in fact significantly reduce and contain the infection spread.

The choice of *ɛ* is based on this effective reproduction number $R_{\mathrm{eff}}$ in the situation where there are no restrictions, so that all mobility is allowed. $R_{\mathrm{eff}}$ is calculated as the dominant eigenvalue of the next generation matrix (NGM) [22]. This calculation can be found in §D in the electronic supplementary material. For $R_{\mathrm{eff}}=1.25$, we have *ɛ* = 0.0238.

#### 2.3.1. Initialization of the model

To run our model, we further need to decide where the infections are located at the start of the time horizon. We call this the *model initialization*. We initialize the model by choosing the number of people that reside in each compartment for each municipality. We distinguish two kinds of initializations: *synthetic initializations* and *historical initializations*, the latter based on data from the Dutch National Institute for Public Health and the Environment (RIVM).^1^ The synthetic initializations provide insight in the spread of the infection based on our SEI_2_R_2_ model, and the performance of the various regional sub-divisions, while the historical initializations show to what extent these insights generalize to practical settings.

For the synthetic initializations, we start by choosing the number of exposed inhabitants for each municipality and set the remainder of the populations to susceptible. In a deterministic approach, these numbers do not need to be integers. In a stochastic setting, non-integer values are rounded (see §B in the electronic supplementary material). For the spread of the initial exposures, we consider two extreme cases. In the first case, a *fixed fraction* of each municipality's population is set to exposed. In the second case, we place *all* exposed individuals in one municipality. After the exposures are set, we simulate the model for 10 days so that sufficiently many exposures have led to infections and new exposures, because in practice this is the point when the outbreak is detected and the measures are introduced. This leads to two synthetic initializations that we will refer to as *evenly distributed* and *concentrated*, respectively. The evenly distributed initialization mimics a widespread outbreak while the concentrated initialization represents a superspreader event. See §C in the electronic supplementary material for details.

**Figure 2.**
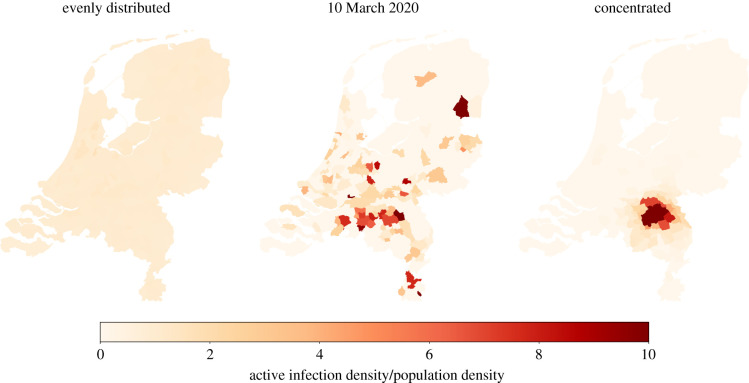
Difference in concentration of infectious people after initialization. The model is initialized with either evenly distributed, historical, or concentrated distributions of active infections. For the synthetic initializations, 1000 people are exposed and 10 days are simulated. The active infection density equals the number of infections within the municipality divided by the national total number of infections. The population density equals the municipality population divided by the total population.

For the historical initialization, we assume that daily updates are available on the cumulative number of confirmed infections in each municipality. Based on these daily cumulative numbers, the number of active tested infections can be estimated by the difference between the cumulative numbers at time *t* and one infectious period before that. The number of untested infections can be found using the assumption that a fraction *a* of the population is tested. The number of exposed individuals can be found by looking ahead one latent period. A more detailed description of these initializations can be found in the supplementary material (see §C). Figure 2 shows how the infections are distributed throughout the country in each of these initializations.

**Figure 3.**
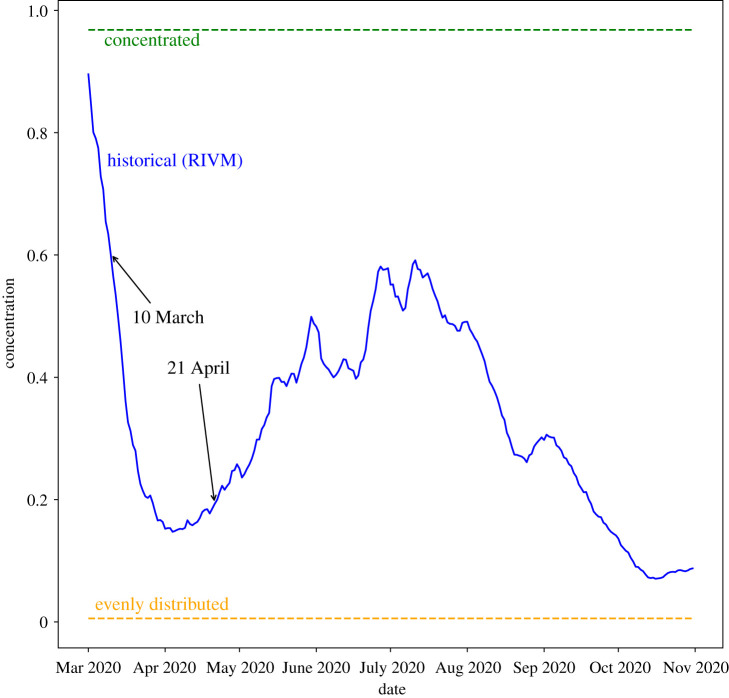
The concentration values corresponding to distributions of infections according to the RIVM data over time. The dotted lines show the concentration levels of the synthetic initializations. Data obtained from https://data.rivm.nl/covid-19/.

To quantify the concentration of the infections, we compare the distribution of the infections over the municipalities to the distribution of the population over the municipalities. For this, we introduce an entropy-type measure. Let $p_{i}^{\inf}$ denote the fraction of infectious individuals that live in municipality *i* and let $p_{i}^{\;\mathrm{pop}}$ denote the fraction of the population that lives in municipality *i*. If the infections would be distributed evenly, then these two distributions would be equal. We measure the concentration of the infections by $1-e^{-D_{\mathrm{KL}}}$, where *D*_KL_ denotes the Kullback–Leibler divergence from the infection distribution ${\left(p_{i}^{\inf}\right)}_{i\in A}$ to the population distribution ${\left(p_{i}^{\;\mathrm{pop}}\right)}_{i\in A}$. Note that this measure does not depend on the total number of infections. When each municipality has the same percentage of infected individuals, the concentration will equal 0. On the other hand, when all infections are located in the same municipality, then it will be close to 1. This allows us to quantify the concentration of the infections for our initializations on a scale from 0 to 1. Synthetic initializations are close to 0 and 1, respectively, while historical initializations have intermediate values. The concentration values of initializations based on the historical data obtained from RIVM are shown in figure 3. In the beginning of the epidemic, there were a few local outbreaks, resulting in a high concentration. As the outbreak became more widespread, the concentration went down, reaching a minimum at the beginning of April. The period after April is characterized by small local outbreaks, leading again to higher concentration values.

### 2.4. Optimization

The quantification of $Q_{\gamma ,H}(D)$ in equation (2.1) is computationally intensive. Firstly, because it involves computing the number of infections at the end of the time horizon and secondly, because the number of possible divisions D is enormous. It is therefore not feasible to find the global optimum. Therefore, we will resort to heuristic optimization methods. To do this, we will iterate the Louvain algorithm [23], which can find partitions that are local optima with respect to the following manipulations [24]: moving a single element from one set to the other and merging together two sets. This algorithm is able to optimize a wide range of functions over partitions [25]. However, each re-evaluation of the score involves running a simulation in our setting, and this method is computationally expensive if the initial division of atomic regions A consists of many small elements. For example, at the finest sub-division possible in our setting, municipality-level, the optimization takes more than 24 h, based on the 380 municipalities in the Netherlands. To improve on this, we define coarser sub-divisions as starting points for the optimization procedure. Instead of letting Louvain operate at the level of single municipalities, we will start from an initial division of the municipalities into sub-regions that will not be further divided by the algorithm. This way, Louvain is guaranteed to result in a division that cannot be improved upon by merging two regions or by moving one sub-region to another region [24]. There are multiple ways to choose such initial divisions. One can use existing administrative divisions such as the twelve provinces of the Netherlands or its 25 security regions. Such administrative divisions may already be known by the public, making it easier to enforce restrictions based on them. A disadvantage can be that such regions have not been defined with an outbreak of an infectious disease in mind and therefore do not pose natural boundaries to transmission. Neither have they been defined on the basis of economic activity. We can also use other criteria to find other initial divisions, for example based on behaviour of individuals that relates to transmission or to economic activity. Human mobility may be a good indicator for both of these. We give two criteria for obtaining such initial divisions.

#### 2.4.1. Mobility regions

In network science, the objective of community detection is to partition the nodes of a network into groups that are more highly connected internally than externally [26]. Community detection has been applied to mobility networks [27,28], resulting in divisions into regions that are coherent with respect to mobility.

Currently, the most popular community detection method is to optimize a quantity called modularity, which computes the weight inside the communities minus the expected weight for a random network with the same weights. For mobility data of the kind that we rely on, the modularity of a division $D=\{D_{1},\ldots ,D_{\left|D\right|}\}$ is given by

$${\mathrm{modularity}}_{\eta }(D)=\frac{1}{M(A,A)}\sum_{D\in D}\left[M(D,D)-\eta \frac{M(D,A)M(A,D)}{M(A,A)}\right],$$

where $M(A,B)=\sum_{i\in A}\sum_{\;j\in B}M_{ij}$ is the mobility between the atomic areas in A⊆A and in B⊆A, and *η* is the resolution parameter, which controls the granularity of the found communities [29]. Larger values of *η* result in divisions consisting of more regions.

**Figure 4.**
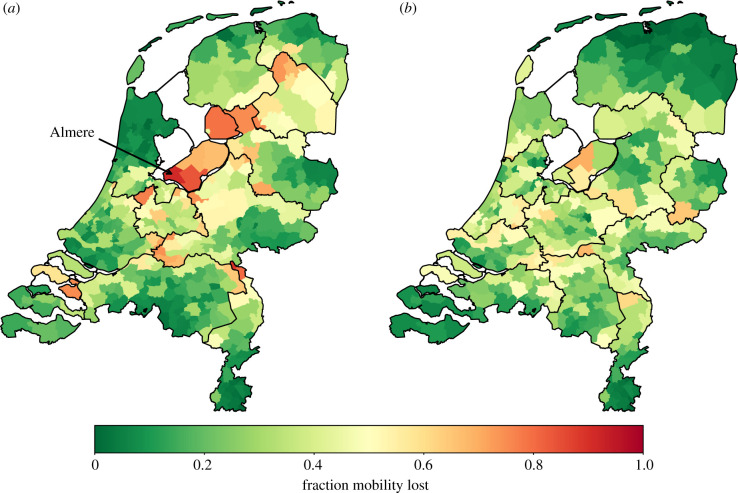
Each municipality is coloured based on the percentage of mobility to destinations outside its region. (*a*) Provinces. (*b*) Mobility regions found by modularity optimization with *η* = 2, chosen such that both divisions consist of 12 regions.

We iterate the Louvain algorithm [23] to optimize modularity. We will refer to regions obtained by this optimization as *mobility regions*. We note that the number of mobility regions that result from applying this method for a given resolution parameter is in general not known beforehand. Therefore, we apply this method for a variety of resolution parameter values to obtain divisions consisting of different numbers of mobility regions. For example, by trial and error it was found that the choice *η* = 2 resulted in a division into 12 mobility regions, so that this division has comparable granularity as the division into the 12 provinces. A comparison of these two divisions based on mobility is shown in figure 4. We see that these mobility regions are indeed more coherent in terms of mobility. In particular, Almere is in the same mobility region as Amsterdam, but in a different province. Mobility regions may reflect the economic and non-local transmission activity of the citizens in a better way. However, they cannot be tailored to the status of the epidemic.

#### 2.4.2. Adaptive mobility regions

**Figure 5.**
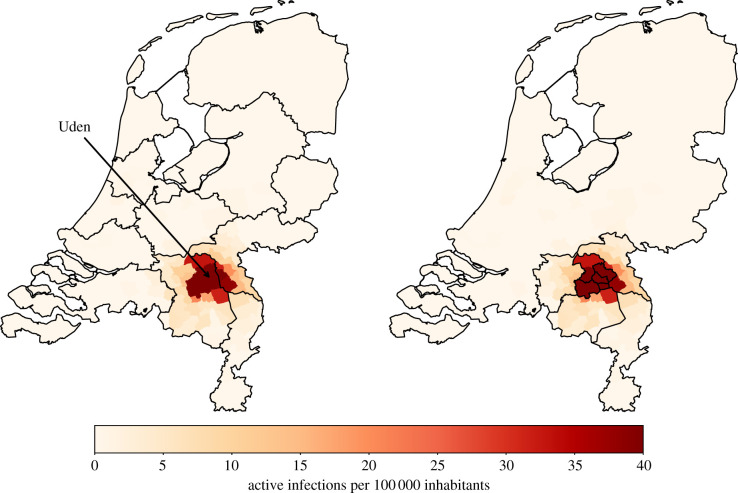
The mobility regions (for *η* = 2) on the left versus the adaptive mobility regions (for *ζ* = 3000) on the right. The colours denote the number of active infections *per capita* for the concentrated initialization. For the colorized figure, we refer to the online version of this article.

The running time of our optimization algorithm depends heavily on the number of sub-regions in our starting division. Note that for the mobility regions, the resolution parameter controls the resolution of the division globally. However, high resolution is mostly needed around locations where a lot of infections occur. We next introduce a modification to the modularity function of the previous section to obtain initial divisions that have a higher resolution near such critical locations. Infections are due to meetings between infectious and susceptible individuals. Therefore, a good heuristic would aim for a division that separates infectious individuals from susceptible ones as much as possible. For a region D⊂A, let *I*(*D*), *S*(*D*) denote the number of infectious and susceptible individuals living in *D*, respectively. We maximize the function

$${\mathrm{adaptive}\;\mathrm{modularity}}_{\zeta }(D)=\sum_{D\in D}\left[M(D,D)-\zeta \frac{I(D)S(D)}{N}\right],$$

where *ζ* is a resolution parameter that, similarly to the *η* parameter of modularity, determines the granularity of the obtained division. In particular, by varying *ζ*, the method results in divisions with different numbers of regions. Despite the fact that *ζ* has a similar role to the *η* of the mobility regions, it does have different dimension and differs by an order of magnitude. Again, the Louvain algorithm is iterated for optimization. We call the resulting regions *adaptive mobility regions* because the resolution is locally adapted to the state of the epidemic. Figure 5 compares a division into mobility regions to a division into adaptive mobility regions for the concentrated initialization. We see that the adaptive mobility regions indeed have a higher resolution around the critical area of the superspreader event in Uden.

### 2.5. Evaluation of divisions

Given values for the trade-off parameter *γ* and the time horizon *H*, we can compute the value $Q_{\gamma ,H}(D)$ to assess the quality of some newly obtained division D. However, by itself this abstract value has no clear interpretation. We can obtain insight by comparing the quality of D to the quality of existing alternative divisions. These existing divisions are referred to as benchmark divisions. There exist administrative choices to divide the country into regions, such as provinces and security regions. We consider the following benchmark divisions for the situation of a widespread outbreak:

— no restrictions (minimum restrictions);
— disallow movement between provinces;
— disallow movement between security regions; and
— disallow movement between municipalities (maximum restrictions).

Note that minimum restrictions maximize mobility and infections while these are both minimized by maximum restrictions. For a superspreading initialization in a single municipality, we also consider the following alternative benchmark divisions:

— isolation of the municipality;
— isolation of the security region of this municipality; and
— isolation of the province of this municipality.

Given a value for the trade-off parameter, we can assess whether the division that is obtained by optimizing the objective for this trade-off value indeed outperforms the benchmark divisions.

**Figure 6.**
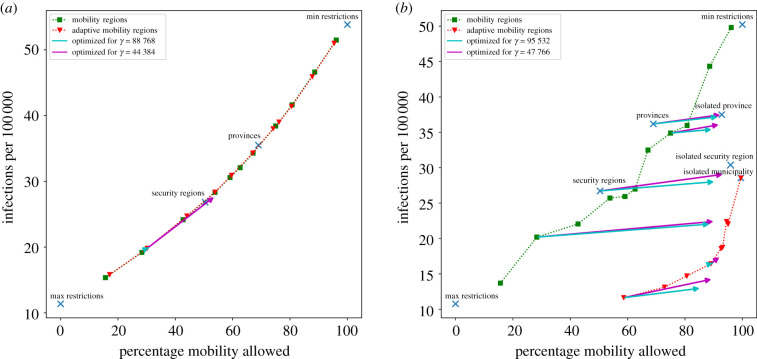
Evaluation of different divisions for the evenly distributed (*a*) versus concentrated (*b*) initialization. The balance of infections and mobility within a horizon of 30 days after imposing a division is shown. Blue crosses represent benchmark divisions. Green squares represent mobility regions, red triangles represent adaptive mobility regions; the parameter values *η* and *ζ* were chosen to obtain divisions that allow various levels of mobility. Arrows point from the starting point of the heuristic optimization to its result. We only show a single arrow for the evenly distributed initialization to avoid cluttering the figure. The used values for *γ* are shown in the legend.

**Figure 7.**
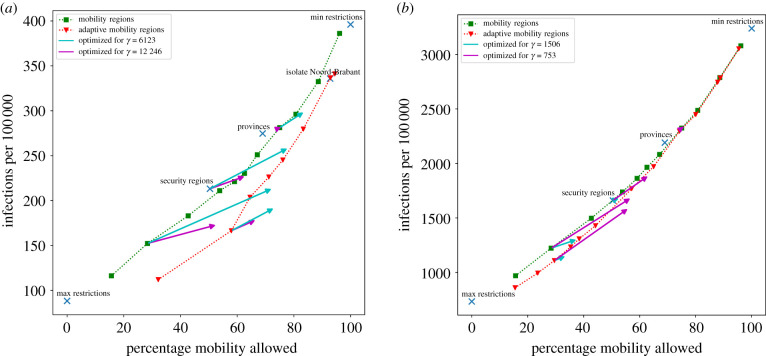
Evaluation of divisions for initialization based on historical data of 10 March (*a*) versus 21 April (*b*) 2020. A horizon of *H* = 30 days was chosen. For our mobility regions and adaptive mobility regions, the parameter values *η* and *ζ* were chosen to obtain divisions that allow various levels of mobility. The used values for *γ* are shown in the legend.

Finally, given a set of divisions $D_{1},\ldots ,D_{k}$ and a horizon *H*, we can plot M(D;H) and G(D;H) for each division, resulting in plots such as in figures 6*a*,*b* and 7*a*,*b*. In these plots, when a division is plotted to the right of a benchmark division, it is more favourable in terms of mobility, while a lower vertical position indicates fewer infections. When a division is such that both are the case, we can say that a division *dominates* the benchmark division: for any value of the trade-off parameter, it will be favoured over the benchmark division. Note that it is not possible to dominate the minimum and maximum restrictions benchmarks, since they achieve maximum mobility and minimum infections, respectively.

Given a set of divisions $D_{1},\ldots ,D_{k}$ and a choice for the trade-off parameter and the horizon, the division with the highest objective value can be chosen. Obviously, the resulting division is not guaranteed to be the global optimizer of the objective as this would require comparing an enormous amount of divisions. Therefore, the quality of such choice depends on the quality of the candidates $D_{1},\ldots ,D_{k}$.

## 3. Results

For various initializations, we perform the optimization described in §2.4 and simulate each of the strategies. Then we evaluate the results based on the objective defined in §2.2. Throughout this section, we consider a time horizon of *H* = 30 days. For each initialization, we apply the optimization method to various initial divisions. The initial divisions will be the administrative divisions (provinces and security regions) and mobility-based initial divisions (mobility regions and adaptive mobility regions for various values of *η* and *ζ*). Recall that we refrain from advising a specific value for the trade-off parameter since it is a political choice. However, in this section, we will consider some values for *γ*, but these are intended only to demonstrate the methodology. We will consider two values for *γ*: the value γ* for which maximum and minimum restrictions have equal objective value (i.e. $Q_{\gamma^{*}}(D_{\min})=Q_{\gamma^{*}}(D_{\max})$ where $D_{\max}=D_{\min}$ are the divisions corresponding to maximum, respectively, minimum restrictions) and twice this γ*. Note that this value γ* is uniquely defined since $Q_{\gamma ,H}(D)$ is a linear function in *γ*. We choose these values for the trade-off parameter rather than fixed constants because constant trade-off parameters may lead to minimum restrictions for one initialization and maximum restrictions for another. This choice of γ* is dependent on the initialization and ensures that there is a non-trivial division that outperforms both maximum and minimum restrictions. We start with the synthetic initializations and draw a few observations. Then, we see to what extent these observations generalize to the historical initializations.

### 3.1. Results for synthetic initializations

We consider an evenly distributed initialization where 1000 people are initially exposed. The resulting mobility and infections from each of the divisions is shown in figure 6*a*. We observe a monotonically increasing relation between infections and mobility. This may be explained by the fact that infections grow with the reproduction number, which is linear in the contact rate, which is in our model in turn linear in mobility. This trend is based on data points between 5000 and 30 000 infections, which is a rather small difference. The exponential-like trend is unlikely to hold for long time horizons. This will depend on the speed with which local saturation in contacts starts to influence transmission potential.

For the points shown in figure 6*a*, the division that was obtained by applying our optimization method to our mobility regions (*η* = 16) resulted in the highest objective value for the trade-off parameter γ*. However, this division only performs marginally better than other divisions on this part of the curve. Furthermore, with respect to a trade-off parameter of 2γ*, maximum restrictions perform best among the divisions shown.

Compared to provinces and security regions, our mobility regions heuristics can be used to tune the granularity of the divisions. This allows one to find the optimal balance on this exponential-like curve with respect to the objective function for given values of the trade-off parameter and time horizon. We observe no divisions which have significantly lower infection numbers in combination with as much mobility as in benchmark divisions. From this, we conclude that the amount of mobility that the division allows plays a larger role than the specific way in which this mobility is chosen.

When infections are not evenly distributed, the performances of the different divisions shift drastically. Figure 6*b* shows the performances of divisions for the concentrated initialization. In this initialization, 1000 people are initially exposed within the Dutch municipality of Uden (figure 5). The mobility regions optimization does not depend on the initialization of the model so these divisions remain unchanged. The adaptive mobility regions do depend on this initialization. We see that the adaptive mobility regions significantly outperform the mobility regions and the benchmark divisions: they result in fewer infections while allowing for more mobility. When we apply our optimization method starting from a sufficiently fine-grained division, we obtain a division that performs even better: for both choices of the trade-off parameter γ* and 2γ*, the division obtained from applying the optimization to the adaptive mobility regions (*ζ* = 8 × 10^5^) outperforms all the other divisions shown with respect to the objective. This suggests that the proposed approach can provide suitable strategies for containing superspreading events, where the initial infections are highly concentrated. This holds even when we change model parameters; see §D in the electronic supplementary material where we perform a sensitivity analysis.

### 3.2. Historical initializations

Next, we evaluate divisions for an initialization based on historical data. We consider data from 10 March 2020 and 21 April 2020. Figure 3 shows that their concentration values for how infections are distributed lie in between the synthetic cases. Therefore, we expect their results to also be in between the synthetic results.

On 10 March 2020, the Dutch government advised all citizens of one of the twelve provinces to stay home. At this point, the number of reported infections was low and their distribution was far from even. 21 April was during the Dutch lock-down period, and further along in the first wave of the outbreak, where the infections were more evenly distributed.

Figure 7*a*,*b* show the evaluation results of different divisions. In figure 7*a*, we add a local lock-down of the province containing Uden as a benchmark division. It performs almost as good as one of the divisions from the adaptive mobility regions approach. However, at that time, the goal of the Dutch government was to suppress the virus to prevent new infections. From this perspective, only isolating this province is insufficient as it does not lead to significantly fewer infections than doing nothing (Min restrictions).

Based on these two figures, we see that the findings from the synthetic initializations generalize to more realistic scenarios: when the infections have a high concentration our approach finds divisions that lead to relatively few infections while allowing for a relatively large amount of mobility. Consider the marker corresponding to the security regions division in figure 7*a*: it can be seen that our mobility method has found divisions with (i) significantly more mobility, but a comparable amount of infections; (ii) a comparable amount of mobility, but significantly fewer infections; and (iii) more mobility and fewer infections. A similar conclusion can be made for the case of figure 7*b*, though the improvements are smaller because the initialization has a lower level of concentration.

## 4. Conclusion

In a pandemic, restrictions on mobility of individuals are one of the mitigation measures available to local and national governments. Restrictions on mobility between regions will have an effect in reducing non-local transmission opportunities. The downside, however, is that restricted mobility also has potentially strong social and economic repercussions. Given this, decision makers have to reach a balance between wanted and unwanted effects when restricting mobility. Rather than impose restrictions on a national level, which could maximize unwanted effects, options need to be explored for regional measures. We have presented a method to determine a balance between infection reduction and allowed mobility. We evaluate mobility strategies that use any division of a country into regions, allowing movement within them, while disallowing movement between them. In the case of the Netherlands, we have shown that existing (administrative) divisions such as provinces and ‘security regions’ do not reflect the mobility patterns within the country well, and therefore are not a good basis for mobility restrictions. We expect this conclusion to apply also to other countries.

We have quantified the trade-off between economy (equated here with mobility) and public health (equated here by infections) by introducing an objective function that penalizes the amount of allowed mobility by the resulting number of infections as given by equation (2.1). This trade-off introduces a parameter that can be interpreted as the number of movements that we are willing to restrict in order to prevent the occurrence of a single further infection.

The objective function is nonlinear, computationally heavy and hence infeasible to optimize exactly. Therefore, we resorted to heuristic optimization methods that are shown to produce divisions that perform well with respect to the presented objective.

As a proof of concept for the proposed methods, we have used synthetic and historical scenarios with varying concentration of infections. For each of these settings, we have compared how well the heuristics perform, and have compared the divisions obtained in this way to the benchmarks of existing divisions. Figure 6*a* shows that when the infections are evenly distributed throughout the country, the performances of all of the divisions lie close to an exponential curve. Therefore, the granularity of the division is the only relevant aspect in this case. On the other hand, figure 6*b* shows that when the infections are highly concentrated around one municipality, applying the optimization to adaptive mobility regions results in a division that significantly outperforms the others and is able to prevent more infections while allowing for more mobility.

In practice, the spread of the infections will lie between these extremes. We have introduced a formula to quantify the concentration of the infections and figure 7*a*,*b* show that indeed low concentration values lead to results comparable to the case of evenly distributed infections, while higher values indeed behave similarly to the concentrated case. Traditional epidemiological models do not incorporate geography and therefore cannot adequately deal with situations where the infections are not evenly distributed across the country. In this work, we have demonstrated how incorporating geometry leads to mobility-restriction strategies that are better tailored to the situation at hand. Our main conclusion is that such strategies are highly effective when the geometric spread of infections is low (so that the geometry is the main limiting factor), but less effective when the distribution is rather even (so that geometry is fairly irrelevant). Our main innovation is that we have proposed a method to quantify these statements.

We next discuss some possible extensions. Firstly, we note that our model does not take any other measures into account. On the one hand, this makes the model unrealistic, while on the other hand it keeps the model simple and isolates the effects of mobility restrictions, which are our main focus.

Secondly, the way we model mobility is especially adequate for small countries where people tend to return home after a visit to another city, such as the Netherlands. It would be interesting to use our model to analyse and compare countries that share this property. For some other countries, travelling people might stay at their destination for a period of time, instead of returning home at the end of the day. The model could be extended to allow for such behaviour.

Thirdly, in our model, each region has two compartments for Tested and Untested, and each infection goes through one of these compartments. In reality, there will always be a period when the person is infected but not yet tested. Therefore, the model may be made more realistic by letting individuals transition through these compartments sequentially (possibly skipping the Tested compartment). However, estimating the transition rates for this model can be challenging, because of its dependence on the testing policy.

Finally, in this work, we have decided to mostly base parameter choices that are relevant for the infection spread on previous studies. Alternatively, these parameters could be *estimated* by fitting the model to match historic data. By doing so, outcomes of the model may have a better predictive value. Currently, the numbers of infections are only used to compare with benchmark divisions. In particular, we cannot give estimates on how close these would be to the true value. Especially with historic scenarios, the fraction of reported cases and unreported cases is heavily dependent on the testing policy and availability of tests. Comparing RIVM estimates of active infections and reported infections hints that this fraction is indeed not at all constant [30]. In our model, we assume this fraction to be constant in all scenarios, and it would be interesting to investigate the effect of heterogeneity, just like we now focus on the effect of heterogeneity of infections.

## Endnote

1 The data are available at https://data.rivm.nl/covid-19/.

## Data accessibility

This article has no additional data.
